# Nephrin is necessary for podocyte recovery following injury in an adult mature glomerulus

**DOI:** 10.1371/journal.pone.0198013

**Published:** 2018-06-20

**Authors:** Rakesh Verma, Madhusudan Venkatareddy, Anne Kalinowski, Theodore Li, Joanna Kukla, Ashomathi Mollin, Gabriel Cara-Fuentes, Sanjeevkumar R. Patel, Puneet Garg

**Affiliations:** 1 Division of Nephrology, University of Michigan School of Medicine, Ann Arbor, Michigan, United States of America; 2 Wayne State University School of Medicine, Detroit, Michigan, United States of America; 3 University of Toledo, Toledo, Ohio, United States of America; 4 Division of Pediatric Nephrology, Motts Children Hospital, Ann Arbor, Michigan, United States of America; 5 Veterans Administration, VAMC, Ann Arbor, Michigan, United States of America; University Medicine Greifswald, GERMANY

## Abstract

Nephrin (*Nphs1*) is an adhesion protein that is expressed at the podocyte intercellular junction in the glomerulus. *Nphs1* mutations in humans or deletion in animal genetic models results in a developmental failure of foot process formation. A number of studies have shown decrease in expression of nephrin in various proteinuric kidney diseases as well as in animal models of glomerular disease. Decrease in nephrin expression has been suggested to precede podocyte loss and linked to the progression of kidney disease. Whether the decrease in expression of nephrin is related to loss of podocytes or lead to podocyte detachment is unclear. To answer this central question we generated an inducible model of nephrin deletion (*Nphs1*^Tam-Cre^) in order to lower nephrin expression in healthy adult mice. Following tamoxifen-induction there was a 75% decrease in nephrin expression by 14 days. The *Nphs1*^Tam-Cre^ mice had normal foot process ultrastructure and intact filtration barriers up to 4–6 weeks post-induction. Despite the loss of nephrin expression, the podocyte number and density remained unchanged during the initial period. Unexpectedly, nephrin expression, albeit at low levels persisted at the slit diaphragm up to 16–20 weeks post-tamoxifen induction. The mice became progressively proteinuric with glomerular hypertrophy and scarring reminiscent of focal and segmental glomerulosclerosis at 20 weeks. Four week-old *Nphs1* knockout mice subjected to protamine sulfate model of podocyte injury demonstrated failure to recover from foot process effacement following heparin sulfate. Similarly, *Nphs1* knockout mice failed to recover following nephrotoxic serum (NTS) with persistence of proteinuria and foot process effacement. Our results suggest that as in development, nephrin is necessary for maintenance of a healthy glomerular filter. In contrast to the developmental phenotype, lowering nephrin expression in a mature glomerulus resulted in a slowly progressive disease that histologically resembles FSGS a disease linked closely with podocyte depletion. Podocytes with low levels of nephrin expression are both susceptible and unable to recover following perturbation. Our results suggest that decreased nephrin expression independent of podocyte loss occurring as an early event in proteinuric kidney diseases might play a role in disease progression.

## Introduction

The kidney filtration barrier is size and charge-selective and prevents passage of macromolecules into the urinary space. The barrier is comprised of the endothelial cells that line the blood capillaries, specialized glomerular basement membrane and unique epithelial cells called podocytes. Podocytes are terminally differentiated cells that have numerous membrane processes that interdigitate to form the final layer of the filtration barrier. Though the podocyte intercellular junction is similar to the adherens junction there are a number of proteins that are found exclusively at the slit diaphragm [1]. Mutations in one such protein called nephrin have been identified to be responsible for a rare disease called congenital nephrotic syndrome of the Finnish type where the developing fetus has proteinuria *in utero* [1,2]. Nephrin is a transmembrane protein that belongs to the immunoglobulin superfamily. Human mutations in nephrin result in developmental failure of the foot processes and the slit diaphragm [2]. Nephrin deletion in mice reproduces the human phenotype mice are born with global foot process abnormalities and fail to survive beyond 48–72 hours following birth [3].

It has been reported by a number of investigators that there is decreased expression of nephrin in human proteinuric kidney diseases at both mRNA and protein level. Similar observations have been made in animal models of glomerular disease [4–11]. Kidney sections from patients with diabetic kidney disease show decrease in nephrin expression that is proportional to disease severity [12]. Streptozotocin-induced diabetic mice have evidence of two distinct populations of glomeruli; large diseased glomeruli that have low expression levels of nephrin and normal sized healthier glomeruli with normal levels of nephrin expression [13]. These studies suggest that decrease in nephrin expression may be an early maker of glomerular disease and might play a role in progression. The observed decrease in nephrin expression could be due to decrease in podocyte density as podocyte depletion has been observed in glomerular diseases and is associated with progression.

We generated an inducible model of nephrin gene deletion in an adult mouse in order to examine the effect of nephrin reduction when podocyte depletion is yet to occur. Following induction nephrin expression decreased by 75–85% within 10–14 days but surprisingly small amount of nephrin persisted at the slit diaphragm for 20–22 weeks. Podocyte density remained unchanged for 4–6 weeks after induction despite the dramatic decrease in nephrin expression. The mice developed proteinuria at 6–8 weeks of age and survived for 20–22 weeks. Kidney sections at 20 weeks post-induction show histological features of focal segmental glomerulosclerosis. Though the small fraction of nephrin that persisted was able to maintain the junction for a relatively long duration it was not enough for recovery (recovery in the rest of manuscript refers to reversal of foot process effacement) in both protamine sulfate and nephrotoxin model of podocyte injury. These results support ongoing role of nephrin in maintenance of the podocyte intercellular junction while suggesting a prominent role of nephrin in reconstituting the junction following injury. Decrease in nephrin expression impairs the ability of podocytes to recover following injury making them susceptible to detach. The development of FSGS, a disease strongly linked with podocyte depletion supports our contention that nephrin depletion makes podocyte susceptible to detachment. Our results support the observation that a decline in nephrin expression without a change in podocyte density impairs podocytes ability to recover after injury and potentially influences disease progression.

## Material and methods

### Antibodies and immunoblotting

Purified rabbit polyclonal antibodies against nephrin, p-nephrin antibodies have been described previously [14–17]. **β**-actin and synaptopodin antibodies were obtained from Sigma-Aldrich. Proteins were extracted from plasma membranes in RIPA buffer (PBS containing 0.1% SDS, 1% Nonidet P-40, 0.5% sodium deoxycholate and 100mM potassium iodide). HALT phosphatase and protease inhibitors were added to the RIPA buffer. Lysates were sonicated with 3–4 bursts of 5–10 seconds using Branson sonicators at the lowest setting.

### Generation of *Nphs1* flox mouse

The targeting vector was constructed based on the sequence information (ENSMUST00000006825)) from the mouse genome databases. The *nphs1* gene is located on chromosome 7, harbors 30 exons over 27.5 kbps. We designed the targeting vector with two loxP sites flanking exon 5 (residues 20805–21301). The purified construct DNA was introduced into the pro-nuclei of fertilized oocytes from 129SV/J mouse at the Transgenic Mouse core facility at the University of Michigan School of Medicine. Southern blot analysis confirmed germ line transmission of the *nphs1* flox allele in WT/flox mice. DNA from the ES cells were digested with *MfeI* and probed with ^32^P radio-labeled ATP. A band of 11.9 kb seen in wild type ES cells changes to a band of 6.8 kb following homologous recombination. The WT/flox mice were then crossed with 129SV/J mice expressing FlpE recombinase to remove the neomycin resistance selection cassette. The offspring containing the *nphs1* floxΔneo allele, confirmed by Southern blot analysis, were bred to establish a homozygous line of mice with two flox alleles (*nphs*1fl/fl). Primers used to distinguish between wild type and *nphs1*^flox^ allele are: 5’ -CTGATCTGGGGTAGCGAGAG- 3’ and 5’–GCTTGGACCTTCCACACATT- 3’. In the presence of *Cre* recombinase, exon 5 of the *nphs1* flox allele is deleted, causing a frameshift mutation immediately after exon 4 resulting in premature stop codon that would stop translation after the first 196 amino acids. The protein is truncated at the second extracellular IgG domain without the transmembrane and intracellular domain.

### Generation of podocyte specific deletion of *Nphs1*

*Nphs1*^fl/fl^ mice were crossed with nphs2-cre mice where *cre*-recombinase is driven by the previously described podocyte-specific podocin promoter (*nphs2*) [18]. For experiments, *nphs1*^fl/fl^, podocin-creTg/+ (homozygous for the floxed nphs1 allele and heterozygous for the podocin-Cre) mice were used. In order to generate inducible deletion of nephrin, *Nphs1*^fl/fl^ homozygous mice were breed with previously described *nphs2*-iCreER(T2) mice obtained from Dr. Danesh Farhad (Baylor University, Houston, TX) [19]. These mice express tamoxifen-inducible *cre*-recombinase under the podocin promoter. *Nphs1*^fl/fl^/nphs2iCreER(T2) mice were fed tamoxifen chow (Envigo TD130858, Tamoxifen 500 mg/kg) for 10 days to induce deletion. Control mice received regular chow. To confirm robust expression of *cre*-recombinase we used the previously described double–fluorescent cre-reporter mouse [STOCK Gt(ROSA)26Sortm4(ACTB-tdTomato,-EGFP)Luo/J; obtained from The Jackson Laboratory, Bar Harbor, ME] that expresses red fluorescence before and green fluorescence after *cre*-mediated recombination [20]. This *cre*-reporter mouse line contains a loxP–flanked membrane–targeted Tomato Red (mT) followed by membrane–targeted EGFP (mG). The targeted vector was designed with a CMV enhancer/chicken β–actin core promoter (pCA) driving expression of the loxP–flanked, N–terminal membrane–tagged tdTomato protein (mT) followed by a polyadenylation signal. Immediately distal to the second loxP site is an N–terminal membrane–tagged EGFP sequence (mG) itself followed by a polyadenylation site. *Cre*–mediated recombination results in deletion of the mT cassette, allowing expression of GFP located downstream in *cre*-expressing cells or tissues. Nphs2-iCreER(T2) mice were initially bred with the double-fluorescent Cre-reporter mice (referred to as the mTmG mice in the text) to generate mTmGfl/fl, *Nphs2*-iCreER(T2) mice.

### Mouse kidney perfusion

Perfusion of mouse kidneys with protamine sulfate was carried out as described previously [17]. Briefly, mice were anesthetized with a combination of ketamine and xylazine; mouse core temperature was monitored with a rectal probe, and animals were maintained at 37°C throughout the procedure using a heating pad apparatus. Kidneys were perfused with solutions maintained at 37°C through the abdominal aorta at a pressure of 120 mmHg and an infusion rate of 12 ml/min (42). Perfusion was carried out with HBSS for 2 min, followed by perfusion with protamine sulfate (2 mg/ml in HBSS; Sigma) for 15 min. This was followed with wash out using HBSS for 5 min. For effacement recovery, after perfusion with protamine sulfate, mice were perfused with heparin sulfate (1 mg/ml in HBSS; Sigma) for 15 min (41) followed with wash out using HBSS. Glomeruli were isolated using graded sieving as described previously [14,16,21].

### Nephrotoxic nephritis model

Nephrotoxic serum was a kind gift from Dr. David Salant (Boston University, Boston, MA). These experiments were performed as described previously [15,22–24]. In brief, 4 week old *Nphs1*^fl/fl,icre(ER)T2+^ mice and control *Nphs1*^fl/fl,icre(ER)T2-^ littermates were injected retro-orbitally with sheep anti-rat glomerular lysate (NTS) or sheep IgG (control) at a concentration of 1.5 mg per mouse (approximately 25 gm. BW). Urine was collected daily over 8 days post-injection. For transmission and scanning EM analysis, tissues were perfusion fixed as described previously [21].

### EM and slit diaphragm frequency analysis

Preparation of mouse kidneys for scanning and transmission EM were performed by standard methods. For scanning EM 20 glomeruli from each kidney section were analyzed with AMRAY 1910 Field Emission Scanning Electron Microscope (SEM) at the University of Michigan Microscope and Image Analysis core facility (MIL). For transmission EM (TEM), kidney sections were examined by the JEOL USA JEM-1400Plus electron microscope at the University of Michigan Microscope and Image Analysis core facility (MIL). Slit diaphragm frequency using TEM was assessed by counting the number of foot process junctions per micron of the basement membrane using Image J software. Kidneys from at least 5 animals were analyzed in each experimental condition.

### Study approval

All animal studies were approved by the University Committee on the Use and Care of Animals Institutional Review Board at the University of Michigan School of Medicine. All work was conducted in accordance with the principles and procedures outlined in the National Institutes of Health Guidelines for the Care and Use of Experimental Animals.

### Immunofluorescence and immunohistochemistry

Kidneys were either perfused fixed or immersion fixed with freshly depolymerized 4% paraformaldehyde in 0.1M phosphate buffer, embedded in paraffin and sectioned at 3–4*μ*m using a Leica Microtome (RM2255; Leica Microsystems). Slides were de-paraffinized in xylene, exchanged twice in 100% ethanol followed by hydration through a series of graded alcohols. Epitopes were retrieved using Retrieve-All antigen unmasking solution (Covance SIG-31910), at 92°C to 94°C for 3–4 hours in a water bath, cooled at room temperature, residual aldehydes were quenched in 0.1% sodium borohydride in Sorenson phosphate buffer for 15 minutes and permeabilized in 0.1% Triton X-100 for 10 minutes. Slides were blocked at room temperature for half hour with 10% host serum of secondary antibody. Sections were incubated overnight at 4°C with appropriate dilution of primary antibody (anti-Nephrin antibody at 1:500, anti-Podocin antibody at 1:400) in 1% BSA. Subsequent to washing steps, slides were developed with fluorescent Alexa dye labeled secondary antibody at 1:100 dilution. For immunohistochemistry, sections were developed using Peroxidase/DAB immunohistochemistry (VECTASTAIN Elite ABC HRP Kit Catalog Number PK-6100 and SIGMAFAST DAB Substrate, Catalog Number D4293, Sigma, St. Louis, MO) using anti-Nephrin antibody according to manufacturer’s protocols. The development of DAB substrate was timed and kept consistent across the samples. Development time was determined by monitoring peroxidase activity in control slides. Sections were counterstained with Gill’s Hematoxylin for staining nuclei but avoided on those slides used for measurements.

### Immunogold electron microscopy

Periodate-lysine-paraformaldehyde (PLP) perfused fixed kidneys were sectioned at 100-μm thickness using a vibratome (Leica Microsystems). Aldehydes were inactivated with 0.1% sodium borohydride in phosphate buffer for 15 minutes. Sections were permeabilized with 0.05% Triton X-100 for 30 minutes, blocked using a combination of normal 5% goat (host) serum, 0.1% cold water fish-skin gelatin, and 1% BSA (Electron Microscopy Sciences, Hatfield, PA). After blocking, slices were incubated overnight with primary antibody (1:500 dilution) in 0.1%–0.2% Aurion BSA-c incubation Buffer (Electron Microscopy Sciences). After thorough washes, specimens were incubated with nanogold conjugated goat anti-rabbit (ultra small, Electron Microscopy Sciences) diluted (1:50 dilution) in incubation buffer for 2–3 hours at room temperature. Sections were later washed, fixed in 2% glutaraldehyde, and processed for silver enhancement per manufacturer’s instructions (Electron Microscopy Sciences). Sections were post-fixed with 0.5% osmium for 15 minutes followed by embedding in resin. Tissue was further sectioned and mounted on 200 mesh copper grids. Specimens were analyzed using a JEOL USA JEM-1400Plus Transmission Electron Microscope at the Microscopy and Image Analysis Core Facility, University of Michigan, Ann Arbor, USA.

### Podocyte number and density

Average podocyte numbers per glomerulus and podocyte density were estimated as previously described [25]. Additional studies for mouse kidney tissue were performed as the method has been previously validated in rat (S1 Appendix). Paraffin embedded sections were de-paraffinized, retrieved using T-EDAT buffer and incubated in methanol containing 3% hydrogen peroxide in order to inactivate endogenous peroxidase activity. Slides were then blocked in 10% goat serum for 30–60 minutes and incubated overnight with anti-WT1 antibody (Abcam: ab89901) diluted at 1/1000 (0.247μg/ml) in incubation buffer containing 0.5% BSA plus 5% goat serum in PBS. Subsequently, slides were developed with a HRP-DAB substrate as per manufacturer’s protocol. (Vectastain Elite ABC kit, Catalog # PK-6101 and SIGMAFAST^™^ 3,3-Diaminobenzidine tablets, Catalog # D4293). Slides were cleared in alcohols and xylenes, mounted with permount (Fisher SP15-500) and scanned to digitalize at 40x magnification using Aperio AT2, Leica Biosystems. Digital images were extracted and analyzed for podocyte profile number, podocyte nuclear diameter and glomerular profile area (S1 Fig). Podocyte number, density and glomerular volume were calculated as described previously [25].

### Nephrin half-life estimation

Intensity of nephrin staining was measured and compared between WT and nephrin-deleted kidney tissues using immunohistochemistry. Animals were sacrificed 2,4,6,8,10 and 16 weeks after tamoxifen-treatment (10 days of tamoxifen chow), kidneys were harvested, fixed in neutral buffered formalin and embedded in paraffin. Time 0 were nphs^fl/fl,Cre+^ animals that were fed regular chow. Sections were developed using Peroxidase/DAB immunohistochemistry using anti-Nephrin antibody according to manufacturer’s protocols. Approximately 50–140 glomeruli from each section were imaged under identical light settings in an unbiased fashion with glomerular samples covering both cortical and medullary regions. Images were imported into MetaMorph Image System software (Molecular Devices, Sunnyvale, CA). Pixel intensities of brown substrate were measured at following settings; Hue 0–50, Saturation 0–250 and Intensity 0–60. Integrated pixel measurements (sum of grey pixels under the area of threshold) for Nephrin were measured for each glomerular profile and averaged. Then ratios between averaged-integrated pixel measurements and average glomerular profile areas were calculated and finally expressed as a percent of control at time zero. A half-life exponential decay curve was plotted after 2 weeks following tamoxifen injections and a nephrin half-life was calculated based on the exponential decay equation.

### ImageJ quantitation and statistical analysis

Data are presented as Mean ± SEM throughout the text unless otherwise specified. The number of experiment performed for each condition has been mentioned in the figure legends. All experiments were performed at least 3 times. ImageJ software (NIH) was used to quantify the fluorescence in at least 10 cells in each glomerulus. 10 or more glomeruli in each experimental condition were examined to generate the data for statistical analysis. Using image J drawing tool an outline was sketched along the cell membrane to measure the fluorescence (For podocytes cells on the periphery of the tuft were selected). A region next to each cell that had no fluorescence was selected for background fluorescence. Corrected total cell fluorescence (CTCF) was calculated using the formula CTCF = Integrated density–(area of selected cell x mean fluorescence of background reading). Statistical comparisons were performed using two-tailed t test or ANOVA where applicable. A value of P≤ 0.05 was considered to represent statistically significant difference.

## Results

### Deletion of nephrin during development results in proteinuria and foot process abnormalities

**Fig 1:**
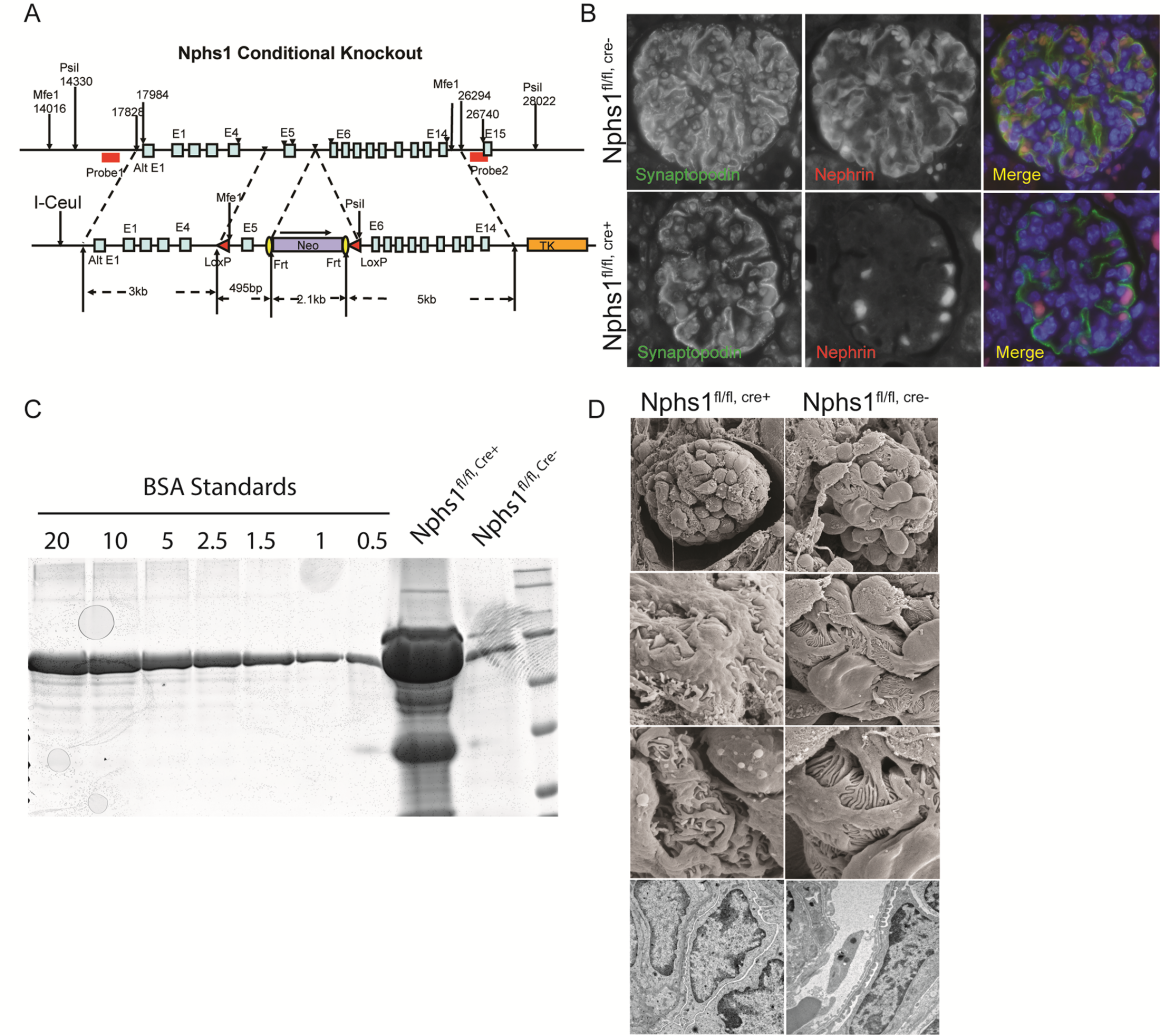
Conditional deletion of nephrin in podocytes. (A) Schematic of the targeting vector containing a neomycin cassette, Frt sites and loxP sites were engineered to flank exon 5 of mouse nephrin. Successful recombination was verified using Southern blot with probes 1 and 2 flanking the nephrin gene. (B) Immunofluorescence images showing absence of nephrin in paraffin embedded mouse kidney section. Synaptopodin staining was used to identify podocytes. C) Coomassie blue stained SDS-PAGE gel showing severe albuminuria/proteinuria in the Nphs1^fl/fl,Cre+^ animals at 2 weeks following birth. BSA standards (0.5–20 μg/dl). (D) Transmission and Scanning electron microscopy images showing podocyte foot process developmental abnormalities following nephrin deletion.

*Nphs1* deletion in germ line has been shown to result in a developmental phenotype where the newborn mice have proteinuria and foot process developmental abnormalities [3]. We generated a mouse with Lox-P sites flanking exon 5 of *nphs1* on chromosome 7 (Fig 1A). Successful recombination was confirmed using Southern blot as well as PCR. Npsh1^fl/fl^ mice homozygous for the flox allele were bred with podocin-cre mice to generate podocyte-specific deletion of nephrin. Animals were born in normal Mendelian distribution and were normal at birth. Deletion of nephrin was confirmed at the protein level by indirect immunofluorescence of kidney sections from wild type and podocyte-specific nephrin-deleted animals (Fig 1B). Synaptopodin was used as a podocyte marker. Since the protein is truncated at the 2^nd^ IgG domain in the extracellular part of nephrin, the translated protein lacking the transmembrane domain cannot be inserted at the membrane. The mice developed massive proteinuria at 1-week of age (Fig 1C). Scanning and transmission EM images of mouse kidney sections show aberrant foot processes in the nephrin-deleted podocytes (Fig 1D). These results confirm that embryonic deletion of nephrin results in the expected phenotype. Podocyte-specific deletion of nephrin resulted in proteinuria at 1 week of age likely as a result of delayed deletion since the *cre*-expression is under the podocin promoter. *Nphs1*^fl/fl,cre+^ mice did not survive beyond 2 weeks of age.

### Inducible model of nephrin deletion

**Fig 2:**
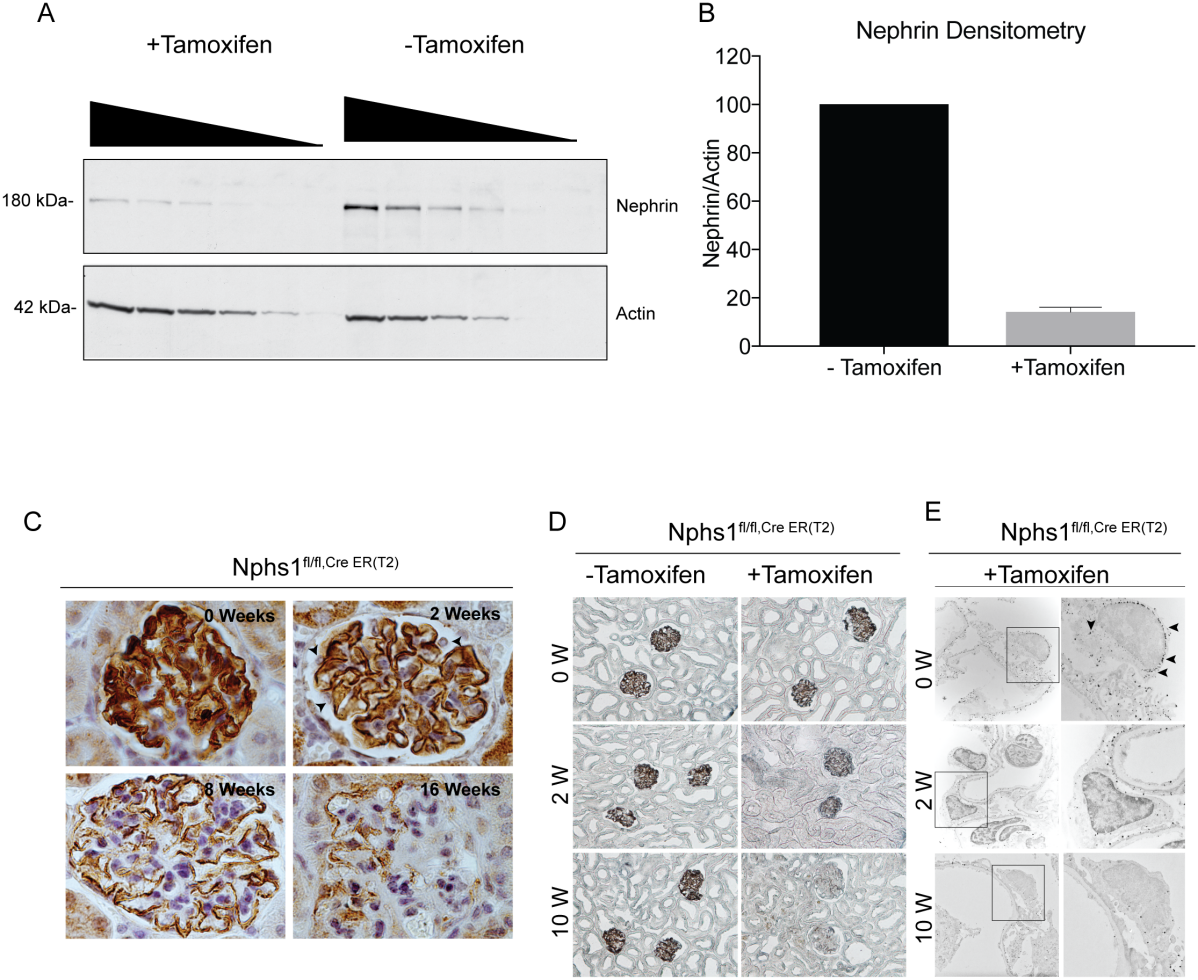
Induced model of nephrin deletion in podocytes. (A) Western blot showing decrease in nephrin in glomerular lysates from Nphs1^fl/fl,iCre(ER)T2^ mice kidneys 14 weeks post-induction with tamoxifen. Decreasing amounts of lysate volume were loaded in each well to avoid reaching the threshold of the x-ray film. Actin is used as loading control. (B) Densitometry showing quantification of the decrease in total nephrin following deletion. Results are representative of 4 separate experiments. (C) Immunoperoxidase staining for nephrin at various time points following tamoxifen-induction. Arrowheads show loss of cytoplasmic nephrin staining in podocytes at 2 weeks. (D) Light microscopy images of kidney tissue sections following silver enhancement of immunogold particles. (E) Immunogold electron microscopy of mouse kidney sections at 2 weeks and 10 weeks showing gold particles (arrowheads) at the slit diaphragm and within the cell body. Gold particles are seen primarily at the slit diaphragm in Nphs1^fl/fl,Cre+^ at 10 weeks. Right panel shows enlarged images of the boxed areas in the left panel. Images were taken at X12,200 and cropped to emphasize the immunogold particles.

In order to study the effects of nephrin-deletion in a mature glomerulus we generated an inducible model of podocyte-specific nephrin deletion using tamoxifen-induced *cre* expression driven by the podocin promoter (*Nphs2*-iCreER(T2) mice) [19]. The expression of *cre*-recombinase using tamoxifen was verified by initially breeding the Nphs2-iCreER(T2) mice with the mTmG double fluorescent cre-reporter mice (S2 Fig). There was robust change in fluorescence from red to green in more than 90% of the glomeruli in a homogenous manner indicating efficient *cre*-mediated deletion of the floxed allele. The *Nphs2*-iCreER(T2) mice were then bred with our nephrin flox mice to generate homozygous nephrin^fl/fl^ mice carrying inducible *cre*–cassette (*Nphs1*^Tam-cre^). Deletion of nephrin using the inducible system was confirmed by Western blotting (Fig 2A). Quantification of the blots using densitometry shows approximately 86% loss of nephrin expression, 14 days after initiation of tamoxifen (Fig 2B). Kidney sections were stained for nephrin using immunohistochemistry at 0, 2, 8 and 16 weeks time points (Fig 2C). At 2 weeks following tamoxifen, nephrin staining is primarily seen at the membrane. There is progressive decline in nephrin staining with focal areas on the membrane showing nephrin at 12–16 weeks. Similar observations were made by immuno-EM (Fig 2D, arrowheads show Nano-gold particles) where gold particles are primarily seen at the slit diaphragm at 8 weeks compared to control.

### A small fraction of nephrin is stable at the membrane

**Fig 3:**
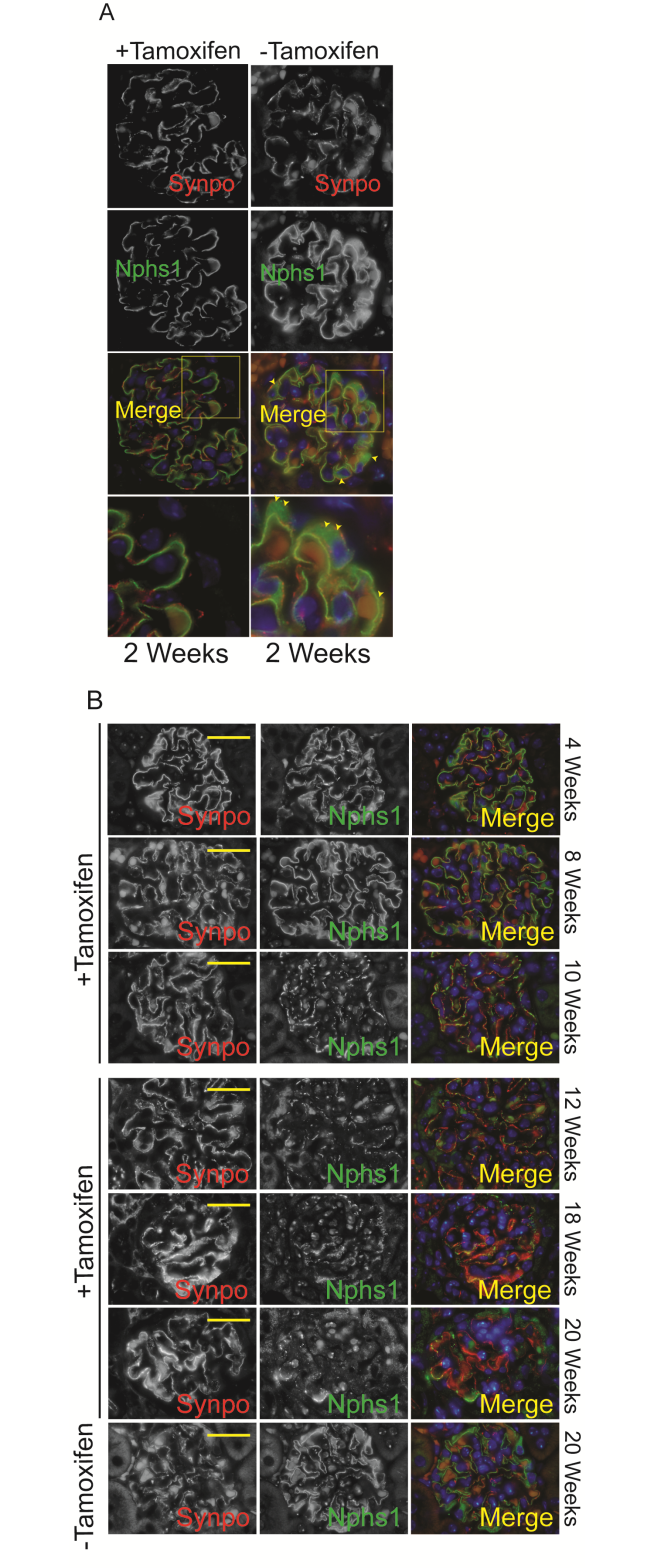
Deletion of nephrin in adult mature glomerulus. (A) Immunofluorescence images showing nephrin and synaptopodin staining at 2 weeks following Cre-induction with tamoxifen. There is abrogation of peri-nuclear nephrin staining following induction (arrowheads). (B) Immunofluorescence images showing nephrin and synaptopodin staining at various time points following tamoxifen. Scale bars, 20 μm.

Kidney sections from *Nphs1* ^Tam-Cre^ mice were stained for nephrin at various time points. Mice that were not fed tamoxifen show nephrin staining along the capillary loops as well as staining in the cytoplasm (Fig 3A, Top Panel: arrowheads). In comparison nephrin staining in the tamoxifen-fed mice is primarily seen along the capillary loops where as the cytoplasmic staining seen in the wild type mice is not evident (Fig 3A bottom panel). The loss of cytoplasmic/peri-nuclear staining would suggest abrogation of newly translated nephrin following nphs1 gene deletion. There is a progressive decrease in the amount of nephrin staining at the membrane with aging, at the same time it appears to be interrupted. Interestingly, nephrin staining is evident for almost 20 weeks following deletion (Fig 3B).

### Deletion of nephrin in a mature glomerulus results in development of FSGS

**Fig 4:**
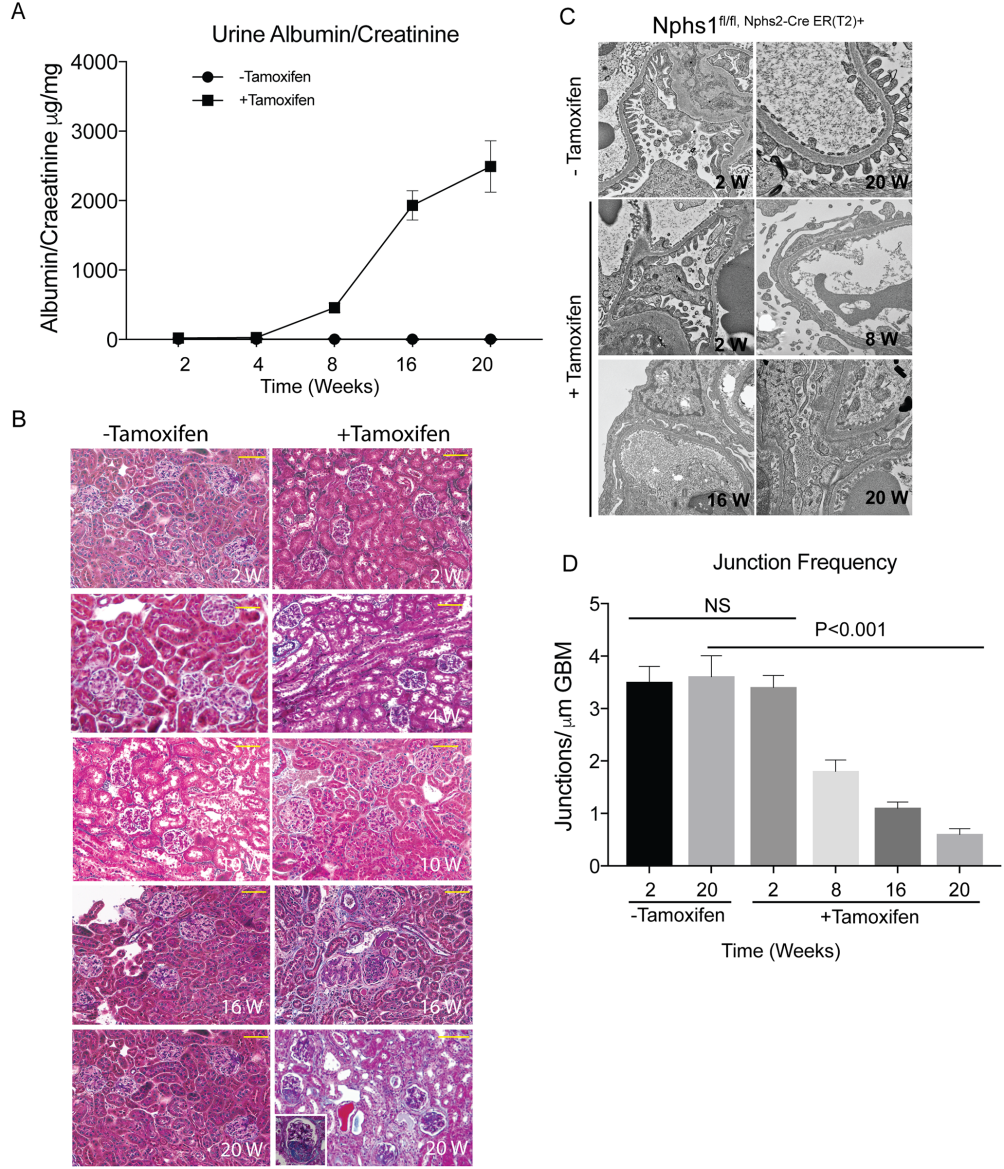
Nephrin deletion in adult mature glomerulus results in focal segmental glomerulosclerosis and proteinuria. (A) Mice with tamoxifen-induced nephrin deletion develop proteinuria at 6–8 weeks following tamoxifen. (B) Masson Trichrome Verhoeff staining of mouse kidney sections at different time points following tamoxifen-induction showing development of focal and segmental glomerular scarring at 16 weeks following tamoxifen. Scale bars, 20μm. (C) Transmission EM images show normal foot process structure at 4 weeks following tamoxifen (Images were acquired at 10,400X and cropped for each panel). There is widespread foot process spreading at 8 weeks following tamoxifen. (D) Assessment of junction frequency shows decrease in the number of podocyte intercellular junction frequency at 8 weeks following tamoxifen. All results are representative of 4 sets of experiments with 5 animals in each group. Data are Mean ± SEM.

**Fig 5:**
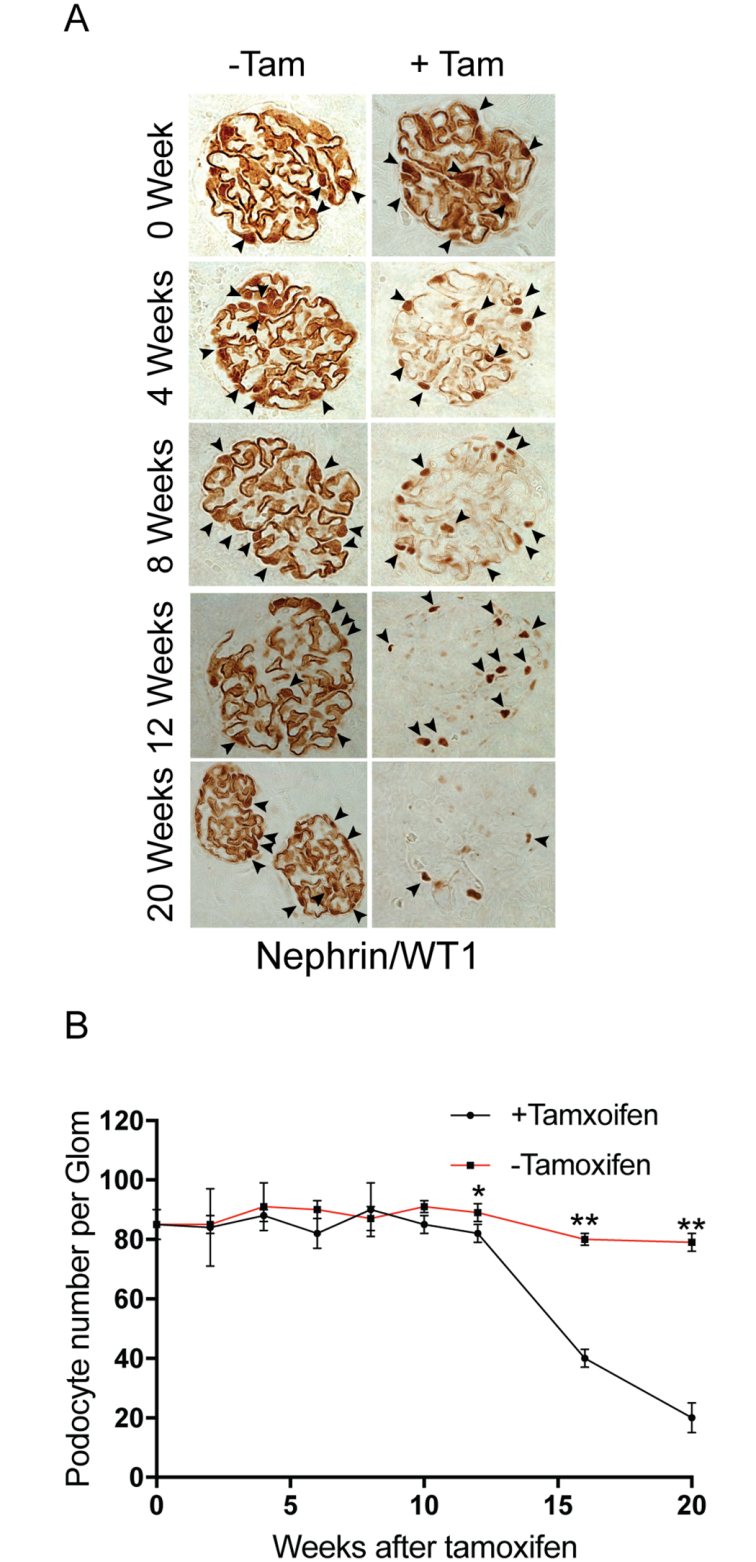
Podocyte numbers remain unchanged for 10–12 weeks following nephrin gene deletion. (A) Immunohistochemistry using nephrin and WT1 antibodies simultaneously shows presence of nuclear WTI (arrowheads) staining despite loss of nephrin in kidney sections at 12 weeks after tamoxifen-induced nephrin deletion. (B) Podocyte counts per glomerulus at various time points in controls and tamoxifen-induced nephrin deletion. All results are representative of 4 sets of experiments with 5 animals in each group. Data are Mean ± SEM.

Urine collected from the *Nphs1* ^Tam-Cre^ mice on a weekly basis shows no evidence of proteinuria at week 4 following induction (Fig 4A). Proteinuria is evident by week 6 and the mice become heavily proteinuric by 20 weeks of age. Mouse kidney sections stained with Masson Trichrome Verhoeff staining shows focal and segmental scarring in glomeruli at 16–20 weeks post-induction (Fig 4B). There is evidence of tubulointerstitial scarring with proteinaceous casts in the tubular lumen. The decline in nephrin expression correlates with progressive proteinuria. Most mice fail to survive beyond 20 weeks following nephrin gene-deletion. Transmission EM shows evidence of preserved foot process architecture at 2 weeks following deletion with progressive spreading of the foot processes that is co-incident with proteinuria (Fig 4C). There is decrease in junctional frequency along the glomerular basement membrane in the tamoxifen-fed animals (Fig 4D). Using immunohistochemistry kidney sections were stained with nephrin and WT1 simultaneously (Fig 5A). Despite loss of nephrin staining along the foot processes, nuclear WT1 staining remained intact at 12 weeks post tamoxifen-induced nephrin deletion. Quantification of podocytes per glomeruli shows no significant change in podocyte number at 10 and12 weeks post-induction (Fig 5B). The podocyte number decrease by 50% at 16 weeks post tamoxifen induction, when proteinuria is evident. Similarly, there is preserved staining for the slit diaphragm protein podocin at 8 weeks post-induction suggesting that the number of podocytes per glomerulus remains unchanged at this late time point (S3 Fig). There is a marked decrease in podocin at 20 weeks post-induction when there is evidence of significant proteinuria as well as development of focal and segmental glomerular scarring.

### Nephrin associated with actin is present stably at the slit diaphragm

**Fig 6:**
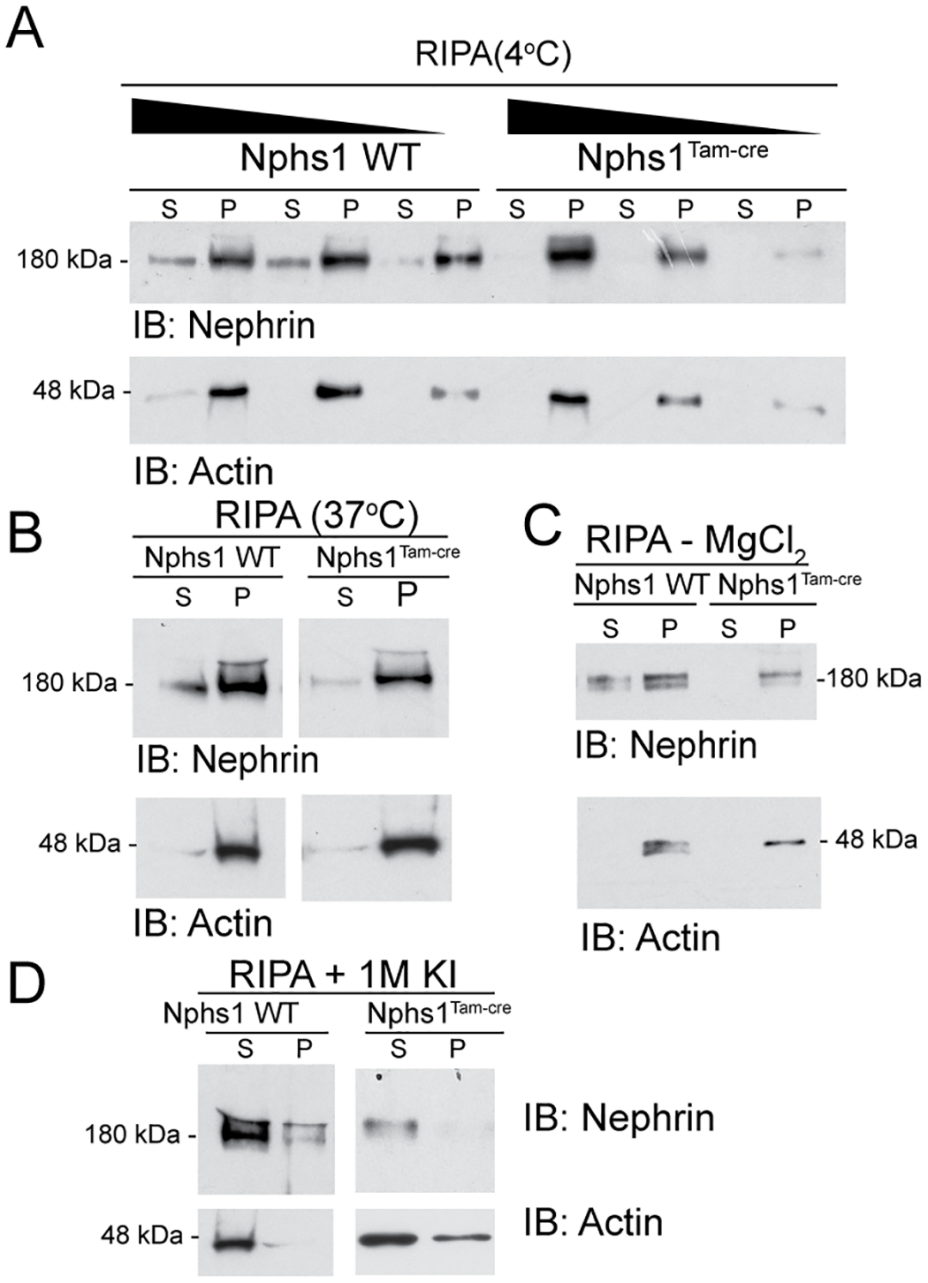
Residual nephrin at the membrane is associated with actin. (A) Isolated glomeruli from wild type and Nphs1^Tam-Cre^ mice kidneys were lysed in RIPA buffer containing 1% Triton X-100 at 4°C. Lysates were resolved with SDS-PAGE gel under reducing conditions and probed with nephrin and actin antibodies. Triton X1000 soluble fraction (S) and insoluble pellet (P) were separated by centrifugation. Nephrin is seen primarily in the insoluble fraction or pellet. (B) Glomerular lysates in RIPA buffer at 37°C showing no improvement in nephrin solubulity. (C) Glomerular lysates in RIPA buffer without 1.5 M MgCl_2_ shows slight improvement in nephrin solubility with prominence of the high molecular weight band in the Nphs1^Tam-Cre^ mice. (D) Glomerular lysates in RIPA buffer containing 1M potassium iodide (KI) show dramatic improvement in nephrin solubility. IB: Immunoblot.

We were intrigued by the stability of a small fraction of nephrin that is seen primarily at the membrane. We hypothesized that this fraction of nephrin is linked strongly with actin at the slit diaphragm. Isolated glomeruli from wild type and nephrin-deleted mice (6 weeks old mice fed tamoxifen for 10 days and sacrificed a week later) were lysed in RIPA buffer containing 1% Triton X-100 alone (4°C and 37°C), RIPA without 1.5M MgCl_2_ and RIPA with 1M potassium iodide. Glomerular lysates were resolved using SDS-PAGE and probed with nephrin antibody (Fig 6). As described previously, nephrin is relatively insoluble when extracted with RIPA buffer containing 1% Triton X100 at 4°C in presence of divalent cation Mg^2+^. Nephrin is enriched in the Triton X100-insoluble pellet that also enriches for actin (Fig 6A) [11,26]. Decreasing amounts of glomerular lysate were loaded to assess the ideal amount needed to analyze the differences. The insolubility of nephrin and enrichment in the pellet, particularly in the nephrin-deleted animals suggests association of nephrin with actin and/or lipid rafts. Extraction in RIPA containing Triton X-100 at 37°C a temperature at which lipid rafts are in liquid state did not improve nephrin solubility significantly (Fig 6B) suggesting nephrin is primarily associated with the actin cytoskeleton. Absence of Mg^2+^ slightly improved nephrin solubility in both wild type nephrin-deleted glomerular lysate where two distinct bands are visible (Fig 6C). Lower amounts of glomerular lysates were loaded for this experiment to easily visualize the two distinct bands. In the nephrin-deleted glomerular lysate absence of Mg^2+^ reveals prominence of the upper higher molecular weight band (Fig 6C, arrow) suggesting enrichment of higher molecular weight nephrin with unidentified post-translational modifications. Addition of the actin depolymerizing agent potassium iodide to the extraction buffer improves nephrin solubility dramatically even at 4°C by releasing it from actin (Fig 6D). Both nephrin and actin are seen in the soluble fraction with relatively small amount in the pellet. These observations confirm that the small fraction of nephrin that remains stable at the membrane is strongly associated with actin.

### Half-life of nephrin at the slit diaphragm

**Fig 7:**
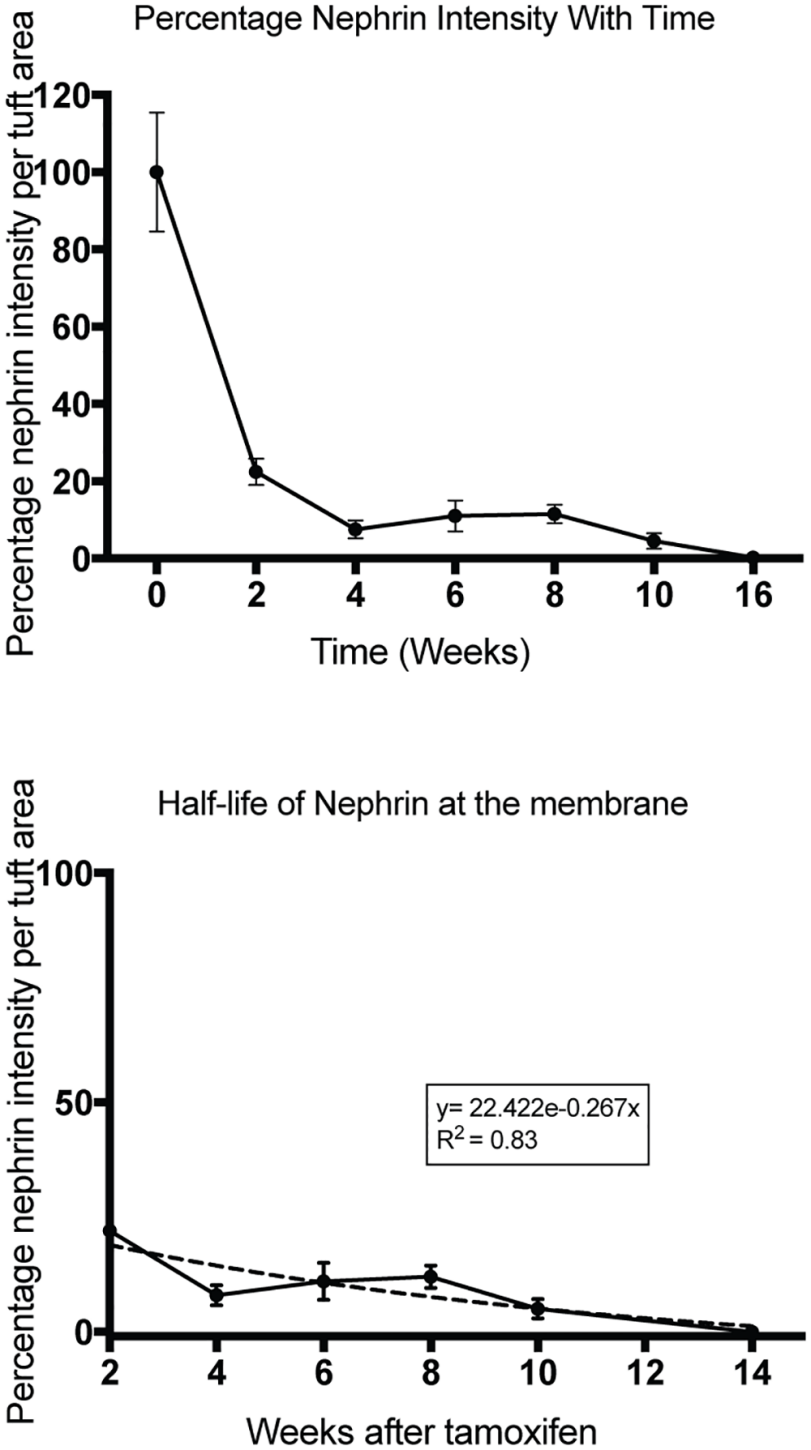
Nephrin has a half-life of 2.59 weeks at the membrane. (A) Mouse kidney sections stained for nephrin using immunohistochemistry were analyzed for nephrin staining intensity at various time points following tamoxifen-induction using MetaMorph image analysis software. (B) Nephrin intensity at time = 0 weeks was plotted as 100%. There is rapid decline in nephrin staining in the initial 2 weeks followed by a slow decline over the next 14–16 weeks. The initial decline represents loss of new-nephrin production and represents nephrin that is primarily within the podocyte cell body. Nephrin staining after 2 weeks is primarily at the membrane (see S4 Fig). (C) Half-life analysis of nephrin at the membrane after the 2 weeks time point. Non-linear regression analysis (dotted line) shows a nephrin half-life 2.59 weeks at the membrane (R^2^ = 0.83). The data is representative of 70–100 glomeruli per kidney from 3–4 animals in each group. Data are Mean ± SD.

Kidney sections from wild type and nephrin-deleted animals were immunostained with nephrin antibody using immunohistochemistry (S4 Fig). The staining intensity was measured using MetaMorph imaging software (Molecular Devices, Sunnyvale, CA). Intensity curves for various conditions were plotted against one another (Data not shown). The intensity of nephrin staining (brown using immune-peroxidase) measured for each animal was in the linear range with lower threshold intensities. With a higher threshold intensity of ≥120, the curves began to flatten off in the WT setting signifying saturation of area measurements while in the knockout the curve rise steeply indicating noise in the system. Thus a setting showing the highest linearity with the maximum intensity was used for measurements. Using an intensity threshold of 60, intensity of nephrin staining around the foot process dropped from 100% at time zero to 30% at 2 weeks (Fig 7A). It further declined with time reaching a nadir at 16 weeks. The primary contributor to the initial rapid decline is the cytoplasmic nephrin, which appears to constitute 70% of total nephrin at any given time point. The remaining 30% of nephrin is relatively stable and present at the slit diaphragm. In contrast to immunohistochemistry the decline in nephrin was almost 85% by Western blotting at 2 weeks post tamoxifen induction (Fig 2A). We used immunohistochemistry to assess total nephrin amount, as the extraction of nephrin was not complete following lysis for Western blotting. In order to calculate the half-life of nephrin we used an exponential trend line applied to nephrin decay curve for nephrin located at the membrane yielding a half-life of 2.59 weeks with an R^2^ value of 0.85 (Fig 7B). The R^2^ value was 0.69 using a linear decay curve with a similar calculated half-life of 2.6 weeks.

### Mature podocytes with nephrin deletion fail to recover from foot process effacement in protamine sulfate model of podocyte injury

**Fig 8:**
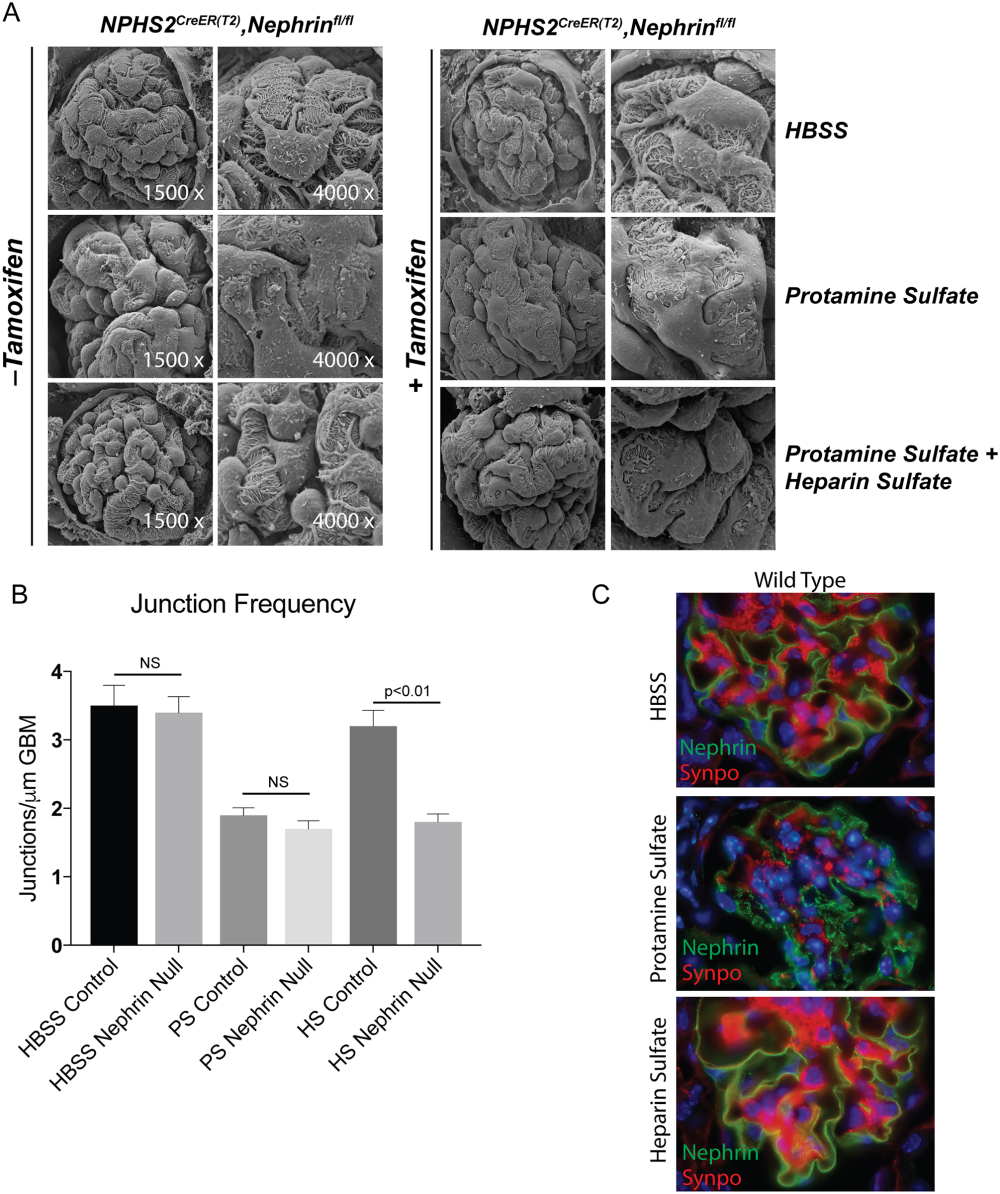
Nephrin-deleted mature podocyte fail to recover following protamine sulfate injury. (A) SEM images of kidneys from 8 weeks old Nphs1^fl/fl,iCre(ER)T2^ mice following protamine sulfate injury. Nphs1^fl/fl,iCre(ER)T2^ mice that received tamoxifen show failure of foot process recovery in response to heparin sulfate following injury with protamine sulfate. (B) Analysis of junction frequency in TEM images show failure of recovery following heparin sulfate in Nphs1^fl/fl,iCre(ER)T2^ mice that received tamoxifen. (C) Immunofluorescence images of wild type animals (Nphs1^fl/fl,iCre(ER)T2,^ tamoxifen^-^) show change in nephrin distribution from the membrane to punctate structures following protamine sulfate injury (middle panel). Nephrin staining is reversed back to the membrane on perfusion with heparin sulfate (lower panel). Synaptopodin (synpo) was used to for double staining the podocytes. All mice used in these experiments were 8–10 weeks old prior to receiving tamoxifen.

In order to investigate the role of nephrin following injury we subjected adult mature mice with inducible podocyte-specific nephrin deletion to protamine sulfate model of podocyte injury. We hypothesized that there would be a partial or abrogated recovery following injury as small amounts of nephrin remains at the slit diaphragm with no evidence of structural abnormalities as well as proteinuria at 4–6 weeks following deletion. 10 week-old animals that received tamoxifen at 6 weeks of age were perfused with protamine sulfate as described previously [17,27]. Kidneys of *Nphs1*^Tam-Cre^ mice with or without tamoxifen were perfused with HBSS (control), protamine sulfate and protamine sulfate followed by heparin sulfate. Animals develop foot process effacement following protamine sulfate infusion (Fig 8A). Interestingly, animals that underwent tamoxifen-induced deletion of nephrin in podocytes (*Nphs1* ^Tam-Cre^) did not recover following heparin sulfate infusion (Fig 8A). Evaluation of podocyte intercellular junction frequency by transmission EM confirmed that both induced and un-induced Nphs1^Tam-Cre^ develop significantly reduced junction frequency after protamine sulfate induced injury (Fig 8B) compared to control (HBSS). The junction frequency in the tamoxifen-induced *Nphs1*^Tam-Cre^ mice failed to recover compared to control (HBSS) as well as un-induced *Nphs1*^Tam-Cre^ following heparin sulfate. Immunofluorescence staining for nephrin shows change in nephrin distribution from the membrane to intracellular vesicle like structures (Fig 8C) following protamine sulfate perfusion. The distribution on the membrane changes from linear to granular suggesting removal of nephrin from the membrane. This is reversed following infusion of heparin sulfate and nephrin distribution returns to the membrane. These results indicate that nephrin’s membrane localization changes following injury and recovery. Similar experiments for the nephrin knock out mice could not be performed, as the amount of residual nephrin was not amenable to staining. It is possible that the small fraction of nephrin that remains stable at the slit-diaphragm following deletion is able to maintain the junction, but is not sufficient for recovery following heparin sulfate perfusion.

### Nephrin-deleted mature podocytes fail to recover following nephrotoxic serum injury

**Fig 9:**
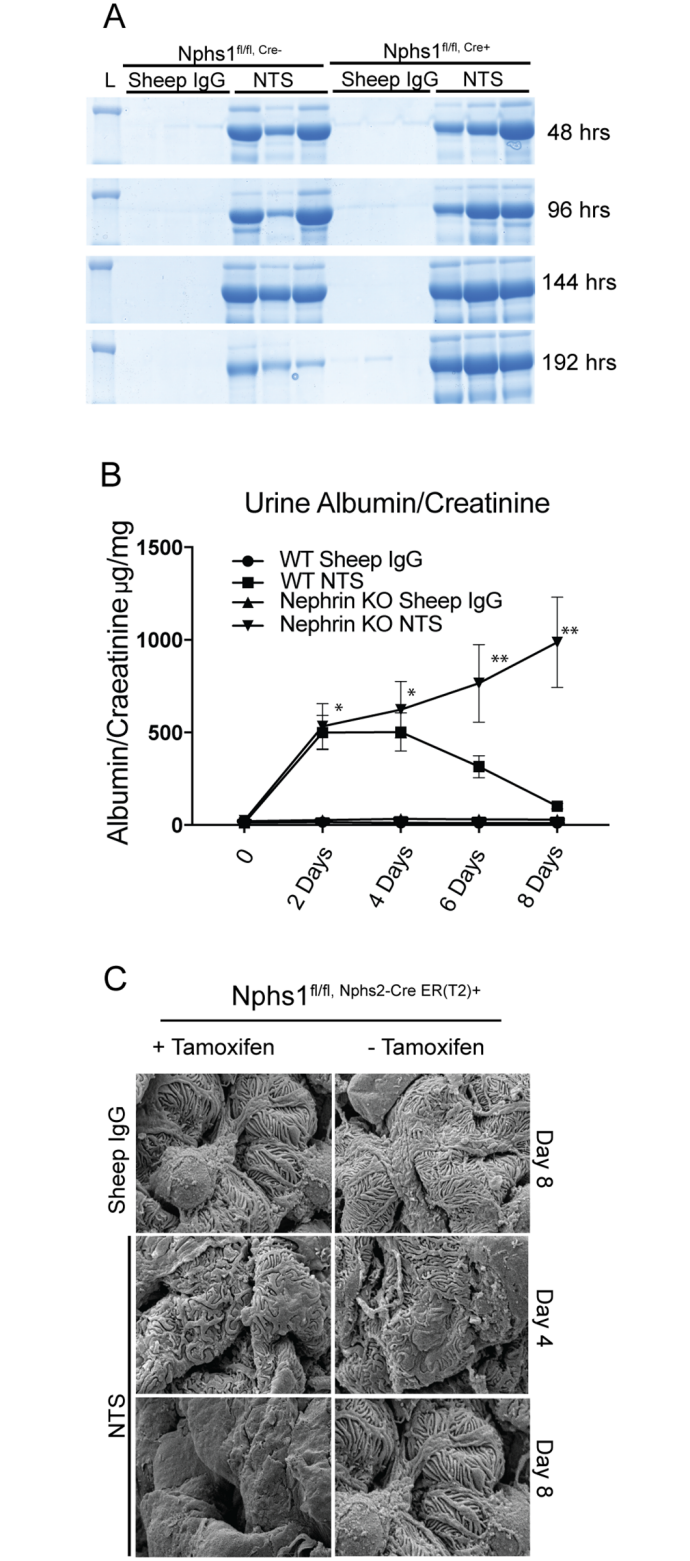
Nphs1^fl/fl,iCre(ER)T2^ mice continue to be proteinuric following NTS injury. (A) Coomassie blue stained SDS-PAGE gel show persistence of proteinuria following NTS single dose administration in Nphs1^fl/fl,iCre(ER)T2^ mice that received tamoxifen. (B) Urine albumin/creatinine ratio from Nphs1^fl/fl,iCre(ER)T2^ mice that were fed tamoxifen and normal chow following NTS injury. Most nephrin deleted mice did not survive beyond 8–10 days following NTS. (C) SEM images from Nphs1^fl/fl,iCre(ER)T2^ mice kidneys with and without tamoxifen induction following NTS injury show failure of recovery of nephrin-deleted podocytes at day 8 compared to control. The data is representative of 4 sets of experiments with 3 animals in each group. Sheep IgG was used as control. Data are Mean ± SEM. *P = Not significant, ** P <0.01, when compared between WT and Nephrin knock out in the NTS group.

To test whether the observations made in the protamine sulfate model could be reproduced in another mouse model of podocyte injury, we used the previously described nephrotoxic serum (NTS) model. Nephrotoxic serum is sheep anti-rat glomerular lysate serum. When injected intravenously, a single dose of NTS produces a dramatic increase in proteinuria within 24 hours and persists for more than 48 hours with subsequent recovery. There is some variability in the response from batch to batch of the antiserum. In our experiments, the proteinuria persisted for 4 days with recovery close to baseline by 8 days in the wild type mice. The proteinuria in nephrin-deleted mice did not improve and continued to rise following injury (Fig 9A and 9B). The nephrin-deleted mice were unable to survive beyond 8 weeks and succumbed to the injury. Scanning EM images show failure of recovery of the foot process spreading in the nephrin-deleted mice that received NTS when compared to control (sheep IgG) (Fig 9C).

## Discussion

The role of nephrin in development of podocyte foot processes is well established and is supported by genetic mouse models and humans with nephrin mutations [2,3]. A decrease in the amount of nephrin present in podocytes has been observed in a number of human and animal models of proteinuric kidney disease including diabetic nephropathy, focal segmental glomerulosclerosis and membranous nephropathy [4–11]. In these diseases, decreases in nephrin mRNA and protein expression at the slit diaphragm inversely correlated with the degree of proteinuria [6,10,12,28]. At the same time, there was no change in the expression of other podocyte specific protein podocin and CD2ap [28]. Diabetic nephropathy has been the focus of a number of different studies that have examined the change in nephrin expression and its correlation with proteinuria [29,30]. Decrease in nephrin, podocin and synaptopodin expression was seen in biopsy samples from diabetic patients [31]. It is interesting that nephrin expression was decreased in the enlarged glomeruli that are presumably undergoing compensatory hypertrophy and represent an early stage of diabetic glomerular disease [31]. Nephrin expression has also been reported to be decreased in glomerular diseases including FSGS, membranous nephropathy and MPGN [4,9,32–35]. Though it has been suggested that the decrease in nephrin expression is a harbinger of podocyte detachment and loss and can be an early biomarker of disease, it could be argued that the decrease in nephrin is primarily due to decrease number of podocytes. Since patient biopsies are only performed when there is evidence of proteinuria or loss of GFR, many of the observations made in kidney sections from patient biopsy samples reflect a time point when significant podocyte loss has already occurred.

In order to separate the issue of decreased nephrin expression from loss of podocyte cell mass we generated an inducible model of nephrin deletion. This model allows us to examine the consequence of decreased nephrin expression prior to the loss of podocytes in the adult mature glomerulus. Unlike the deletion of nephrin during development that results in massive proteinuria and foot process developmental abnormalities, deletion of nephrin in a mature healthy glomerulus resulted in a phenotype that resembles human focal segmental glomerulosclerosis. Following the induction of *nphs1* gene deletion, nephrin expression decreased by 86% by 2 weeks using Western Blot (Fig 2B) and 70% using immunostaining (Fig 9A). There is clear evidence of lack of new nephrin production as the peri-nuclear staining of nephrin seen in controls is abrogated and nephrin staining is seen exclusively at the membrane. The discrepancy in residual nephrin as estimated by Western blotting and immunostaining is likely due to incomplete extraction of membrane-associated nephrin during lysis as it is closely associated with actin at the slit diaphragm. This is the first study to experimentally estimate the half-life of nephrin in podocytes. Based on our results the half-life of nephrin is between 2.5–3.5 weeks and would require 12.5 to 17.5 weeks for nephrin to completely disappear. At the same time there are two different populations of nephrin within podocytes: cytoplasmic nephrin declines rapidly after induction and membrane bound nephrin stays stable and can be seen by immunostaining for 20–22 weeks. Our results using potassium iodide to depolymerize actin support the hypothesis that the nephrin associated with actin is stable and persists at the slit diaphragm. Nevertheless, there is some turnover of the protein present at the membrane as the intensity of nephrin staining declines progressively over 16–20 weeks post-induction and appears to be discontinuous along the membrane. Immunofluorescence shows nephrin shifting from the membrane to intracellular punctate dot like structures that are probably intracellular vesicles following protamine sulfate infusion. Both endocytosis and recycling of nephrin has been shown to occur at the membrane [36,37]. Studies done *in vitro* postulate that nephrin can undergo both clathrin and raft mediated endocytosis and has high rate of turnover [38,39]. Moreover, during podocyte injury, nephrin is mislocalized providing evidence for both endocytosis and recycling [36]. Following tamoxifen induced nephrin deletion a fraction of nephrin appears to be stable at the membrane and is tightly associated with actin. This may represent a small fraction of nephrin that is either not subject to turnover or constantly recycled to the membrane. It is likely that following injury tyrosine phosphorylated-nephrin is removed from the membrane and since newly synthesized nephrin is not available to take its place or recycled nephrin is mislocalized. Though the primary defect in our model is loss of nephrin expression and not podocyte injury, it is likely that ongoing podocyte detachment and loss along with animal growth results in the development of focal segmental glomerulosclerosis. Our study provides experimental evidence that decrease in nephrin expression as observed in a number of glomerular diseases results in progressive loss of podocytes and development of glomerulosclerosis in line with the established podocyte depletion hypothesis of glomerular scarring and obsolescence [40–43].

Previously reported RNA-interference mediated nephrin knockdown in adult mice resulted in proteinuria and foot process abnormalities at 20 weeks whereas short-term knockdown (6 weeks) of nephrin made the mouse susceptible to injury [44]. These observations would support nephrin’s role in recovery of podocyte foot process structure following injury in a mature fully developed glomerulus. It is not surprising that in terms of nephrin expression, studies have shown benefit of angiotensin converting enzyme inhibitors and angiotensin receptor blockers [29,30]. Angiotensin II infusion in SD rats resulted in an initial rise followed by a decline in nephrin mRNA and protein expression when the animals developed hypertension and proteinuria [45]. Angiotensin II was also shown to increase nephrin-β-arrestin2 association and increase in nephrin endocytosis [46]. Renal biopsies from type II diabetic patients who were randomized to receive treatment with perindopril or placebo for the preceding 2 years showed 62% reduction in nephrin levels in patients that received placebo [29]. These patients did not have significant proteinuria or evidence of GFR loss indicating maintained podocytes number though this was not part of the analysis in the study [29]. Similarly, preservation of surface expression of nephrin and reduced proteinuria in diabetic mice by decreasing expression of CIN85 a binding partner of nephrin that mediates nephrin endocytosis [47].

These observations suggest that maintaining nephrin at the slit diaphragm by either increasing its expression or inhibiting its degradation would be beneficial. To our knowledge this is first study that provides experimental data to support observations made by a number of investigators in regards to decline in nephrin expression in various human glomerular disease. It provides unique and new insights into nephrin and podocyte biology that are relevant to human diseases. At the same time our model provides a unique opportunity to test various interventions that increase or stabilize nephrin expression and could potentially be used as treatment for proteinuric kidney diseases.

## Supporting information

S1 Appendix Description of method used to assess podocyte number in mouse kidneys, using WT1 staining. (PDF)

S1 Fig Podocyte counting using WT1 staining. Kidney sections were stained with WT1 antibody using immunohistochemistry. (A) Each glomeruli was identified and numbered using ImagePro premier imaging software. (B) A tracing was applied along the Bowman’s capsule to assess the surface area of the glomerulus. (C) Each WT1 positive nucleus was identified and the nuclear size assessed. See Methods for further details regarding the assessment of podocyte counts.
(TIF)

S2 Fig Cre recombinase induction following tamoxifen is robust and homogenous. (A) Low power magnification immunofluorescence images showing shift in fluorescence from red to green following tamoxifen-induction in the mTmG^fl/fl, Nphs2-iCreER(T2)^ mice. All glomeruli showed the shift in fluorescence suggesting the expression of *cre recombinase* is robust and homogenous. (B) High power magnification images showing individual glomeruli.
(TIF)

S3 Fig Podocin staining following tamoxifen-induction shows preserved podocyte density till 8 weeks. (A) Immunofluorescence images showing podocin (green) and synaptopodin (red) staining at various time points following tamoxifen induction. (B) Quantification of podocin staining using image J software. Results are expressed as integrated density and corrected total cell fluorescence. NS (P value not significant), **P<0.01, ***P<0.001, Error bars, S.E. Scale bars, 20 μm.
(TIF)

S4 Fig Estimation of nephrin half-life. Nephrin staining using immunohistochemistry at various intensities (60%, 90% and 120%) using MetaMorph imaging software. Area being measured is pseudo-colored orange.
(TIF)
