# Supplementary material for: Nephrin is necessary for podocyte recovery following injury in an adult mature glomerulus
Source: PLoS One. 2018 Jun 20;13(6):e0198013. doi: 10.1371/journal.pone.0198013 (PMC6010211; doi:10.1371/journal.pone.0198013)
Supplement: S1 Appendix — (PDF) [file pone.0198013.s001.pdf]

S1 Appendix

Determination of Podocyte number in mouse kidneys using WT1 (Wilms Tumor 1) staining: We initially validated the WT1 antibody as a different anti-WT1 antibody was used previously for rat tissue. Podocytes were double labeled with podocyte specific markers such as Synaptopodin, podocin and nephrin to ensure that every podocyte nuclei was stained by the WT1 and avoid any artifacts. We used both immunohistochemistry and immunofluorescence to compare both methods and found immunohistochemistry to be ideal for our studies. The method described by Wiggins et. al uses single section to predict the podocyte number based on a correction factor. To ensure that our observations were accurate we performed serial sectioning of kidney tissue to count actual podocyte numbers per glomeruli (3-4 animals each condition and at least 10 glomeruli per section were analyzed) in both control and experimental conditions. Data is shown in supplementary figure 3 for each glomerular volume and number of podocytes contained within. Since we imaged using non-confocal methods, and step size of each optical section was unknown, to ensure that this does not result in sampling errors, we measured glomerular volumes in the same animal's kidney tissues using a serial ribbon of 30-35 sections of 3μm section thickness. Glomerular volumes were identical by either method using serial sections for the above animals; we were able to validate the single section approach in mice. After confirming similar results in single and serial sections of mouse kidneys we moved to analyze the data from our experimental conditions. Kidney sections were mounted on glass slides, dried, baked, de-paraffinized, mounted using

Permount® and scanned at 40x. The entire section size was cropped and area measured using ImagePro Premier® (Media Cybermatics, Rockville MD) software. Leica DM IRB microscope was used to acquire images and analyzed using Imagepro Premier® and Metamorph®.
