# Supplementary figures and images for: Nephrin is necessary for podocyte recovery following injury in an adult mature glomerulus
Source: PLoS One. 2018 Jun 20;13(6):e0198013. doi: 10.1371/journal.pone.0198013 (PMC6010211; doi:10.1371/journal.pone.0198013)

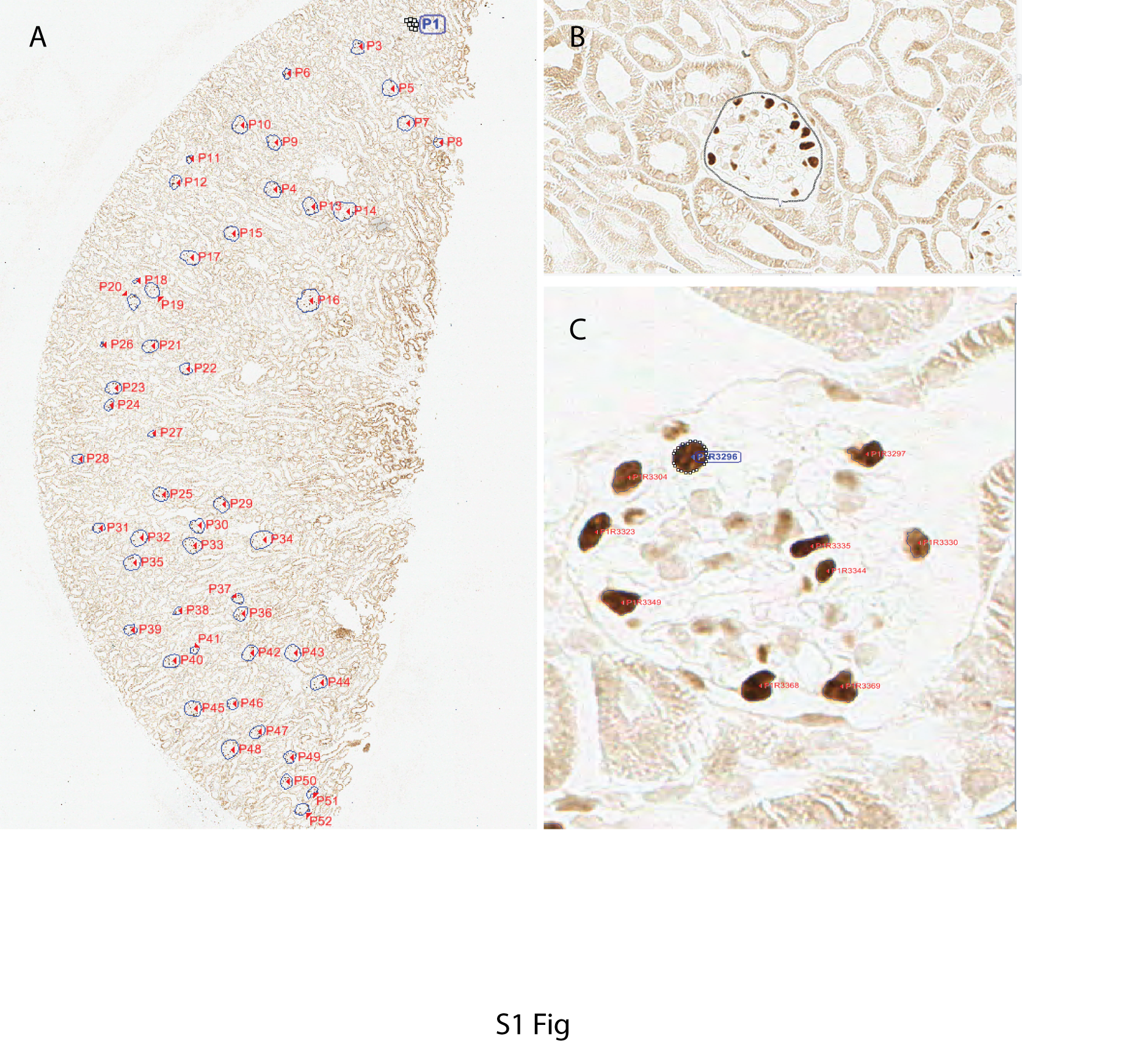

Supplement: S1 Fig — Kidney sections were stained with WT1 antibody using immunohistochemistry. (A) Each glomeruli was identified and numbered using ImagePro premier imaging software. (B) A tracing was applied along the Bowman’s capsule to assess the surface area of the glomerulus. (C) Each WT1 positive nucleus was identified and the nuclear size assessed. See Methods for further details regarding the assessment of podocyte counts. (TIF) [file pone.0198013.s002.tif]

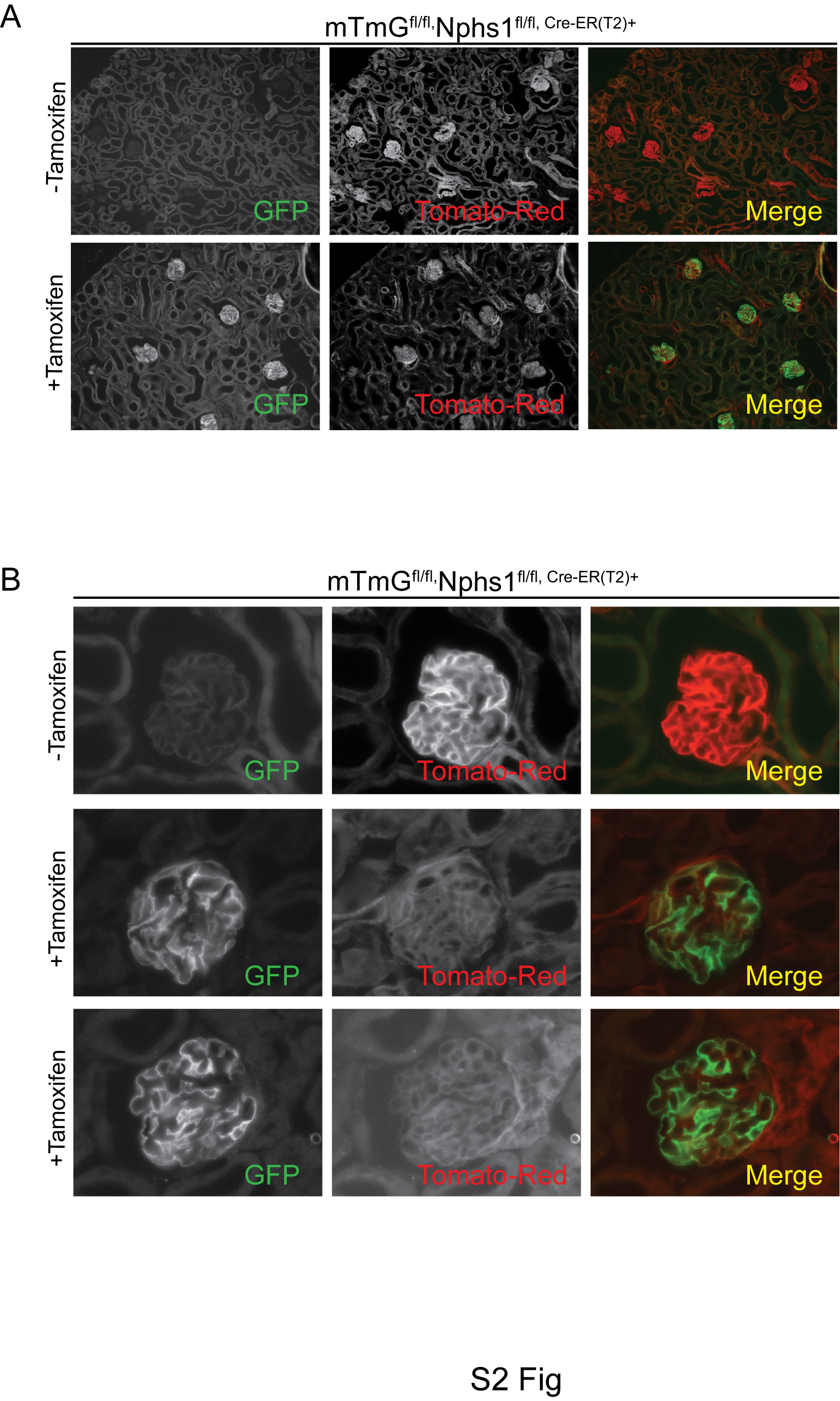

Supplement: S2 Fig — (A) Low power magnification immunofluorescence images showing shift in fluorescence from red to green following tamoxifen-induction in the mTmGfl/fl, Nphs2-iCreER(T2) mice. All glomeruli showed the shift in fluorescence suggesting the expression of cre recombinase is robust and homogenous. (B) High power magnification images showing individual glomeruli. (TIF) [file pone.0198013.s003.tif]

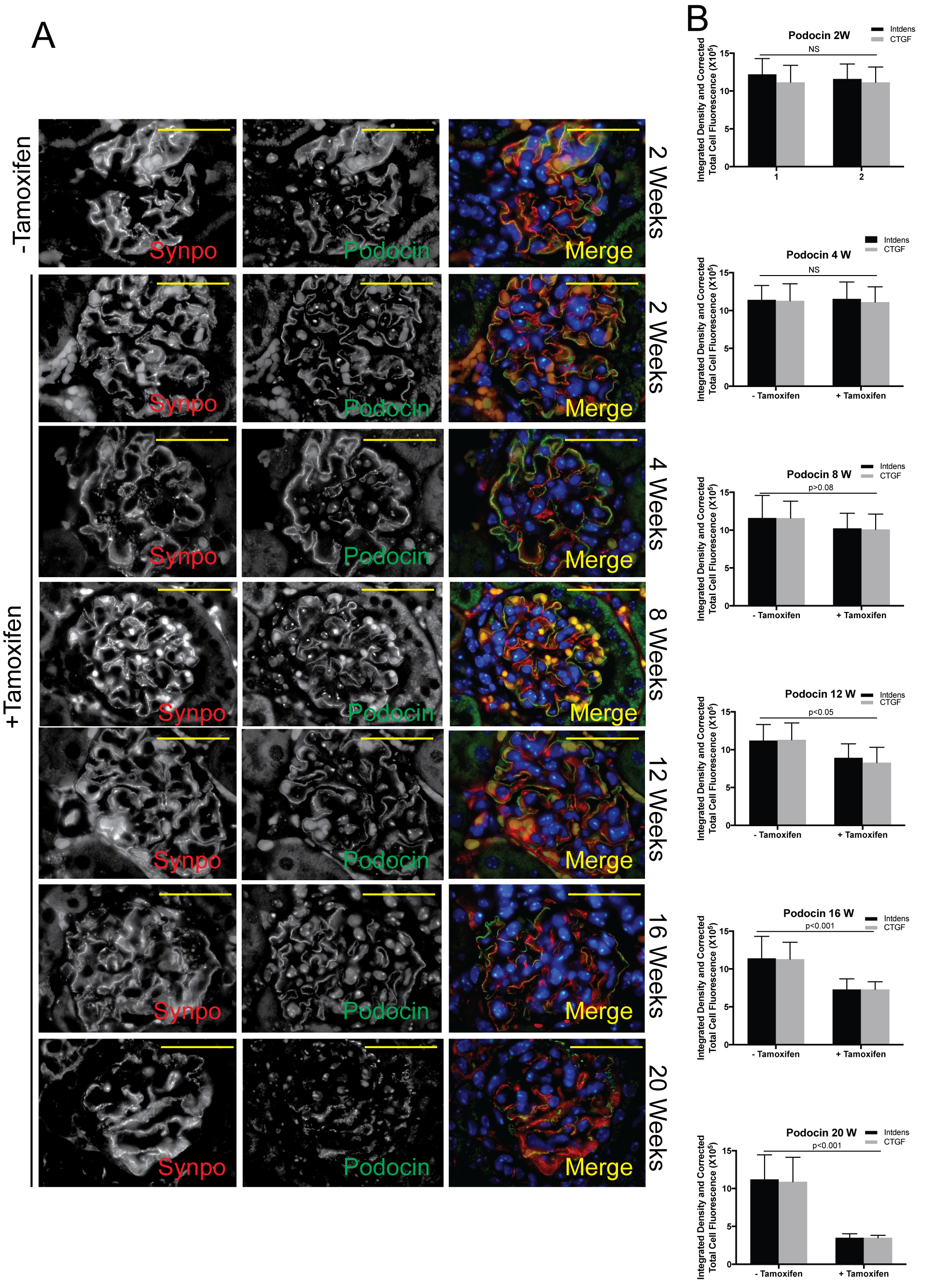

Supplement: S3 Fig — (A) Immunofluorescence images showing podocin (green) and synaptopodin (red) staining at various time points following tamoxifen induction. (B) Quantification of podocin staining using image J software. Results are expressed as integrated density and corrected total cell fluorescence. NS (P value not significant), **P<0.01, ***P<0.001, Error bars, S.E. Scale bars, 20 μm. (TIF) [file pone.0198013.s004.tif]

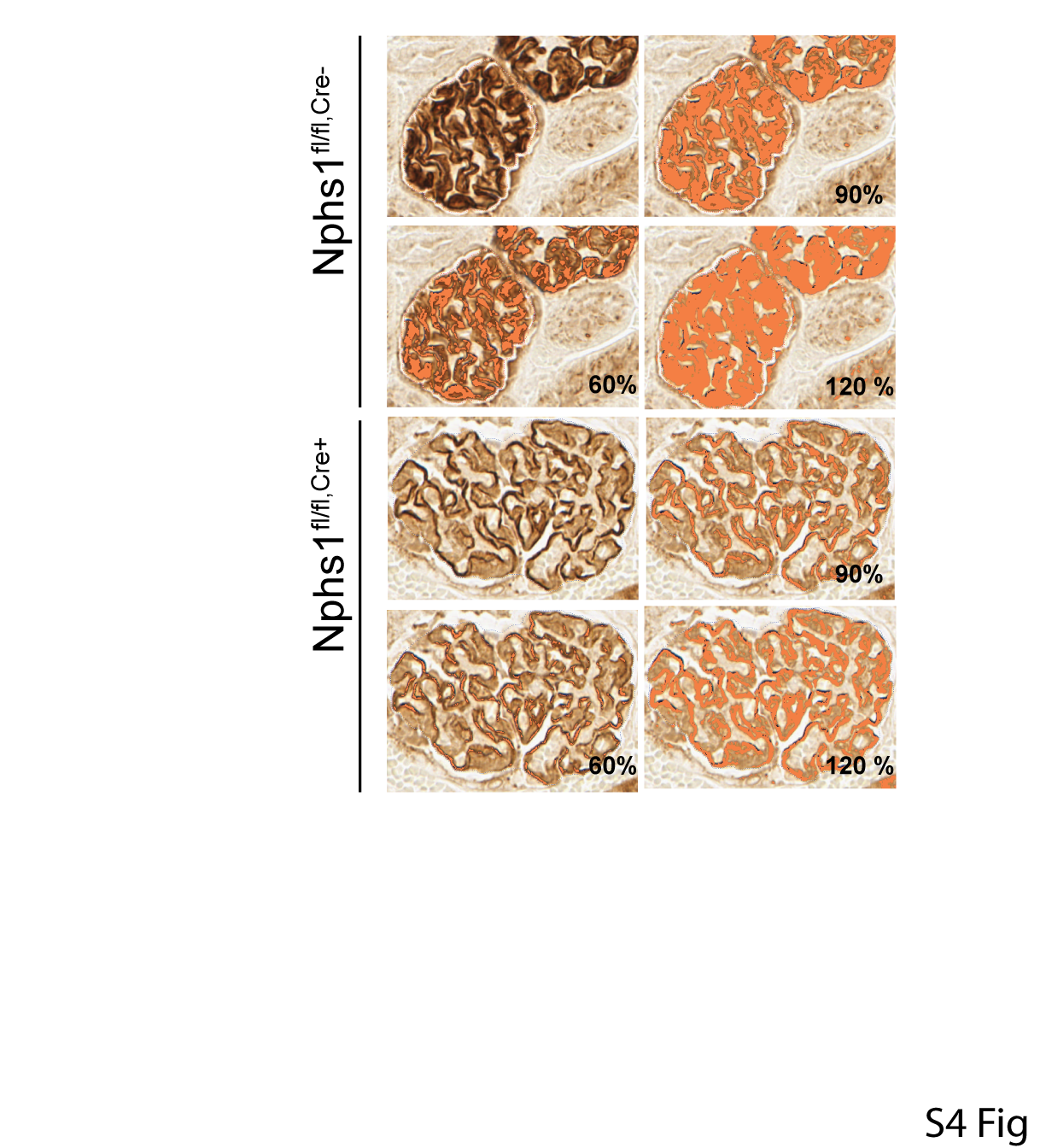

Supplement: S4 Fig — Nephrin staining using immunohistochemistry at various intensities (60%, 90% and 120%) using MetaMorph imaging software. Area being measured is pseudo-colored orange. (TIF) [file pone.0198013.s005.tif]
